# The Prevention of Venereal Diseases

**Published:** 1917-09

**Authors:** H. E. Kleinschmidt

**Affiliations:** St. Louis


### THE PREVENTION OF VENEREAL DISEASES.

**H. E. Kleinschmidt, M.D., St. Louis**

The achievements of preventetive medicine are numerous, yet because they have been brough about chiefly through unsensational methods, the world refuses to be thrilled by the results. There was a time when all inhabitants of England were divided into two classes, those who had smallpox and those who were going to get it. Today smallpox is almost a negligible quantity. Few laymen and some physicians have never heard of Walter Reed, yet his pioneer work in yellow fever has resulted in the saving of millions of dollars and thousands of lives annually. Typhoid fever is so unnecesary that the time will doubtless come when disgrace will attach to the community where it finds a foothold. Infant mortality has been markedly reduced and tuberculosis claims fewer victims per capita since the campaign of education directed against it was inaugurated. These are doubtless the chief reasons why the average life of the American citizen has been increased in the past decade by some three years.

**Tardiness of Attack of Venereal Diseases.**—Disease prevention is largely a matter of individualization if we may use the term; that is, the intelligent application of those remedies best suited to remove the cause of the particular disease attack. For a long time, no one had had the courage to undertake the eradication and control of a certain class of diseases which exacts its fearful toll of human life, domestic happiness, individual efficiency and social progress. The reasons for this tardiness are many. In studying the problem, it was found that this was not merely a question of medicine, but that it involved morals, social relations and time-honored prejudices. Hence the term "social diseases"; indeed it is not improper to speak of the general situation, referring not anly to venereal diseases, but to the causes which were originally responsible for them as a "social disease" just as poverty, crime and some of our distressing economic abuses must be termed "diseases of society." This particular social disease, like most others, thrives in the dark. The "conspiracy of silence" concerning matters of sex and reproduction which probably originated in the Dark Ages was maintained by those who honestly but erroneously hoped to keep the minds of the youth pure and uncontaminated. A general apathy of the public and a hesitancy in even discussing the problem was another serious obstacle to overcome.

Still another unfortunate circumstance obstructing progress was the fact that this class of diseases has been stigmatized as "shameful diseases," when as a matter of fact the disorder is not shameful, but only the immorality which originally caused it. Moreover, this so-called just punishment for disobedience to the laws of nature and society is visited upon the just and unjust alike; virtuous women and innocent children suffering even more severely than the male offender.

And, finally, the campaign for the control and eradication of these diseases has not been made before because the whole subject was shrouded in doubt and mystery. It is only within recent years that the organisms of gonorrhea and syphilis have been discovered while the introduction of salvarsan, the Wassermann reaction and the complement fixation test are still fresh in our memories.

**The New Era.**—However, the dawn of a new day appears. Everyone has noticed a general public awakening in regard to public health measures and especially in relation to the venereal peril. The light of publicity and candor is being turned on, driving before it those evils which flourish best in the dark and secret places. Parents and teachers are beginning to realize that the policy of silence has failed and are substituting correct, clean knowledge, based on biology and physiology and paying careful attention to the development of moral safeguards. Most of these changes may be traced directly to the campaign originated by medical men and have been brought about chiefly by considering veneral disease as a public health problem and by recognizing that the remedy consisted not in a simple system of sanitation, but depends largely on a healthy attitude of mind toward the subject of sex and reproduction. Organized movements fostered by physician, lawyers, business men and others interested in social welfare, have been launched and are now systematically spreading the propaganda. Then with the discovery of the specific causes, known methods of transmission and improved therapeutics, we were ready to undertake the great task of eradicating, at least of limiting the spread of these diseases.

Already a good beginning of the attack on venereal diseases has been made. For purposes of discussion, we might divide the methods of attack roughly into three departments, namely: educational, legal, and medical-social.

**Educational.**—The last few years have seen prudery discredited, and the public, without wincing, has faced the truth. Public sentiment has been jolted into action be learning the startling facts formerly so carefully concealed by the medical profession. It is true that the too common, morbid sensational discussion and treatment of the subject has resulted in harm, but this has been heavily overbalanced by the good accomplished. Publicity must continue and with it an earnest effort to redeem the subject of sex from unclean associations and raise it to its proper high place.

Sex instruction for the young and adolescent is a much debated subject. But this substitution of the question "How shall we teach?" for the query "Shall we teach" indicates a hopeful change of public opinion. Adolescents have a natural desire to learn of the processes by which life is transmitted. Learn they will and it is only a question as to whether that knowledge shall be given them by trained teachers and conscientious parents in a pure and noble way, or whether the information shall come from the street, by way of the foulest mouthed urchin, who is usually the most willing to hand out his misinformation freely and without price.

Education, of course, includes moral training and the establishment of proper social relations. When respectable women cease to condone the indiscretions of youth, so-called, and shall demand the same high standards from men, as those which they themselves observe, fewer "wild oats" will be sown and a cleaner manhood, morally and physically, will be the result, for it is unquestionably true that young ambitious men live up (to a certain degree at least) to the standards set for them by society.

**Legal.**—The materialistic tendency of continental Europe is well reflected in her legal attempts to stamp out venereal disease. Recognizing that the practice of prostitution is the most fertile field for the dissemination of these diseases, repeated and strenuous experiments have been made to regulate and control prostitution, all to no purpose, however, for failure is written in large letters across these various attempts. Those who study the subject of the medical control of prostitution declare the whole system a pitiable farce. Prostitution, especially that portion of it commonly known as "commercialized vice," is susceptible to no compromise. There is only one remedy for this evil and that is suppression. Any partnership of the state with the business of vice is unmoral, unsocial, and a flagrant inconsistency flaunted in the faces of those who have been taught to eschew the very temptations emphasized by the practice.

There has been passed by the legislature of twenty-nine states and by the national Congress for the District of Columbia, a law commonly known as the Injunction and Abatement Law for Houses of Prostitution. It is a most effective weapon in that it strikes a blow not at the unfortunate women concerned, but those who make a profit out of this nefarious business by renting their property at inflated prices for purposes of lewdness. The legal procedure is simplified by placing its operation in the Court of Common Pleas. Briefly stated, it is employed in this way: Any citizen or district attorney armed with proper evidence may appear before the judge who shall declare the place said to be a house of prostitution, a nuisance, and shall enjoin those responsible for its operation and the owner of the property from continuing its use for that purpose. Failure to obey may result in confiscation of real and personal property, and the issuance of a permanent injunction against the house complained of. When intelligently used, the law has resulted not only in the abolition of the segregated district, but has placed an effective weapon in the hands of individual property owners, whose interests might be endangered by the encroachments of the forces of vice. It is regrettable, indeed, that this bill failed of passage in the legislature of Missouri, lacking just two voes, but it is to be hoped that in its next attempt, it will be championed especially by the physicians of this state who can see the advantage of such a law from the purely public health point of view, in addition to the social.

A much discussed legal measure which holds out a certain amount of hope is that of compulsory notification of all cases of gonococcus infection and syphilis. A helpful, honest co-operation between physicians and health departments in this regard would certainly assist in furnishing valuable facts and controlling foci of infection.

By far the most drastic legal measure thus far employed for the control of gonorrhea and syphilis is that enacted by the government of Western Australia. It not only provides for the free diagnosis and treatment of indigent cases, but demands that every person infected place himself under proper treatment until cured. No effort is wasted in reporting the cases by name, but the doctor is held responsible for each case. Heavy penalties serve to enforce these demands, and while it seems harsh and drastic, it must be granted that the rights of the people are best served in this manner. It also stops the advertisement and sale of medicines intended for venereal diseases.

**Medical and Social.**—Briefly then, the program consists mainly of two efforts: (1) building up and strengthening those forces which will best equip the youth in resisting the temptations of sex irregularity, and (2) removing those flagrant temptations which are debliberately planned to entice young men and women. But aside from this, the medical profession has an important part to play. The duty of discovering and treating infected individuals falls on us. If it is the function of a health department to locate cases o contagious diseases, such as diphtheria, smallpox, scarlet fever, etc., fhy not hold the department responsible for locating the active carriers of gonorrhea and syphilis? A few enterprising departments have assumed this task. In New York City the Wassermann test is done without charge and without restriction, and 59,-

614 specimens were examined in 1914, of which 75 per cent. had been supplied by private physicians. Many state health departments offer Wassermann facilities, but it is not generally advertised and properly utilized by physicians. The splendidly equipped department in the state of Pennsylvania has comparatively few requests for this work from the smaller cities and rural districts.

The establishment of free clinics for indigent patients suffering with venereal diseases has passed the experimental stage and is now a proven success. Prejudice against the free treatment of these disorders is rapidly breaking down, especially as those who are responsible for these institutions and interested in the public health realize that this is sound and preventive medicine. In the campaign of education against tuberculosis, numerous dispensaries and clinics were opened for the infected, not because it was hoped materially to reduce mortality from the disease by reclaiming those already suffering with the disease, but because it opened an opportunity for teaching them how to prevent the spread of the disease to others. The same principle applies to the venereal diseases with this important additional factor, to wit: every patient who is properly handled and treated becomes a disciple of the cause. The false notion often maintained by the patient and which probably led up to his misfortune, that syphilis can be easily cured and that gonorrhea is no worse than a bad cold, is replaced by the knowledge of the dire results of these diseases and he is equipped with facts painfully learned at first hand, which he will use in his intercourse with others who are inclined to joke about this serious matter. The Brooklyn dispensary has done pioneer work along these lines. The dispensary for the treatment of syphilis and gonorrhea is conducted with due regard to the privacy and feeling of the patient. He is impressed at the first vista with the idea that his welfare is being considered by those in charge. Records of attendance are carefully kept and a follow-up mailing system employed for those who are delinquent in attendance. Married men are urged to send their wives to the women's department for examination. The health department of New York assists by tracing the cases with active lesions to their places of employment and insisting on continued treatment. In the waiting room of the dispensary, the patient is bombarded with appropriate and striking placards, charts, cartoons, and other messages displayed on the wall all of which emphasize the importance of thorough treatment, and after his consultation with the doctor he is presented with a booklet, giving in concrete form some advice about the prevention and cure of the malady with which he is suffering. Venereal quacks of New York City reap a rich reward by preying on the ills and fears of men, and their advertisements were prominently displayed throughout the city in toilet rooms, public comfort stations and other places. Armed with authority from the health department the Brooklyn dispensary removed these objectionable advertisements and put in their places neat placards giving sound instruction to the venereally infected and offering advice through the department of health. Recently a unique experiment was tried. Coney Island, the favorite summer rosort of New Yorkers, abounds in "health exhibits" conducted by charlatans for the purpose of creating and attracting business. This cue was followed by the sponsors of the Brooklyn Dispensary and a building prominently located in Coney Island was engaged, which had every outward appearance of the regulation quack health exhibit. Once inside, however, the visitor was greeted by an attractive exhibit of sex hygiene of a constructive nature. The results of improper and neglected treatment of gonorrha and syphilis were strikingly illustrated by means of a series of large hand painted plates. Captions such as "What the quack says" contrasted with "What the facts are" were illustrated and elaborated in a most effective manner and a display of what the city department of health and the local Society of Social Hygiene is doing to fight venereal disease was given prominence. During the two months of this exhibit it was visited by more than 19,000 persons, and of those who consulted the attendant 183 were found to be infected.

These were referred to private physicians or to the dispensary situated nearest their homes. A physician was in constant attendance ready to explain the exhibits and advise with those who needed assistance.

**Social Service.**—An almost necessary adjunct of the modern dispensary for venereal diseases is some form of social service. To send out into the community a syphilitic who has taken just enough treatment to rid himself of ouspoken symptoms, is unjust to himself and to the uninfected. The quack accomplishes almost as much. An intelligent social worker, with a sympathetic understanding of human nature and a working knowledge of medicine can multiply the effectiveness of the medical staff wonderfully. In the gyncological department of the Boston dispensary, the social service and follow-up systems are given credit for reducing the amount of wasted work in the clinic from 45 per cent. to 6 per cent. during the year 1915. In one venereal disease clinic, the average number of visits per patient was slightly over seven. After the establishment of a social service department this number was increased to twelve. At the syphilis clinic of Lakeside Hospital in Cleveland, figures are not obtainable, but it is the belief of the superintendent of that institution that the number of visits per patient has almost trebled since the introduction of social service. Even that is only half the story. A trained worker finds situations in the home, the workshop and in private life of the patient which the attending physician would never discover, and which may be of extreme importance in limiting the spread of disease.

**Personal Prophylactic Measures.**—Under the heading of the medical attack on venereal disease, we must not neglect to mention personal prophylaxis. While silver nitrate and calomel ointments used for personal prophylaxis have undoubtedly been successfully employed under adequate supervision, as for instance in the army and navy, the adoption of these measures has not found much favor with the general public. It is not considered wise to create a false sense of security in the minds of immature boys, half-drunken men and careless and unscrupulous prostitutes by the simple application of what most would believe to be an infallible preventive. Unless the application is made within a reasonable length of time, after exposure, by trained physician or carefully instructed layman, and according to the approved method, no reliance can be placed on its effectiveness and it would be wrong to advocate its promiscuous use. Aside from that, however, the chief objection is "based primarly on the public determination to safeguard something it holds far more precious than health, namely, the morals of the community.."

**The Private Practitioner and His Share in the Program.**—Physicians are generally called upon to lead in any movement looking toward the conservation of human life and health, and though it is true, that the main beneficiary, the general public, seems least interested and urgent for improved conditions, it nevertheless devolves on members of the medical profession, whose ideals are supposed to rise far above sordid gain and whose expert knowledge places them in a strategic position for leadership, to engage in this mighty warfare against the venereal peril. The private practitioner can aid in a number of ways; he can supply, without exaggeration, those startling facts which will jostle the public out of its complacency; he can instruct the young and lend sympathetic aid to those who have made of him a father confessor and he can, by his sane conservatism, counteract the damage done by cranks and pedlers of bizarre schemes for the immediate solution of the problem. By urgent and combined demands, doctors can also stir the departments of health into action and convince them of the necessity of furnishing those facilities for diagnosis, advice and treatment which it is the duty of the state to do. Wherever feasible, the practitioner can point out to directors of hospitals and dispensaries the gross injustice of discriminating against gonorrhea and syphilis, and can len his aid in providing adequate treatment for that class "which we have always with us." More attention should be paid in private practice to the scientific diagnosis and treatment of the venereal diseases. Too often such patients receive haphazard and incomplete atteition, greatly to their detriment and that of the public. With the splendid opportunities and improved facilities of recent days, physlcians owe it to themselves and to their patients to give the very best science and art have to offer. Many of us are inclined to shift all responsibility on the patient when he fails to follow prescribed instruction or disappears from our notice. But this spirit of laisse faire is to be condemned, because every physician is in a measure responsible for the public health and he realizes more completely than the careless patient what a menace such a patione is to society. If public institutions find it necessary and practicable to follow up their cases, surely the private practitioner is justified in doing the same without incurring the unjust charge of "pernicious activity" or solicitation. Finally, the physician has the unique opportunity of reaching young men and women in a manner peculiar to the profession. His counsel and advise are sought in matters which are not even trusted with the most intimate associate. Questions relating to the advisability of entering matrimony, the distressing sexual disorders of men, women and children are brought to him with an unbounded confidence in his judgment and sincerity. He dare not violate this secret trust, but is bound, whether it pains him or not, to give freely of his professional advice and human sympathy, looking toward the welfare of the race and the protection of those who seek his counsel.

Let us continue our efforts in this fight and recruit new forces from the lay public. It is not a hopeless strife, neither is it a millennial expectation, despite the time-honored, stale argument of the unprogressives that the social evil has always been with us and therefore always will be. Every important step civilization has ever made has challenged and overthrown this same obstruction.—Journal of Missouri State Medical Association.

